# Lonely but not alone: integrating social alienation and mental health in urban environments

**DOI:** 10.1192/j.eurpsy.2025.1939

**Published:** 2025-08-26

**Authors:** F. Poukhovski-Sheremetyev, I. Gold

**Affiliations:** 1 Department of Psychiatry; 2 Department of Philosophy, McGill University, Montreal, Canada

## Abstract

**Introduction:**

The modern urban landscape is increasingly characterized by the paradox of social isolation in physical proximity. Research consistently reveals a troubling link between social alienation and mental health issues, including a heightened risk for psychosis among vulnerable groups. As cities expand and diversify, understanding and mitigating the detrimental effects of urban alienation becomes crucial. This presentation explores the complex relationship between urban living, social alienation, and mental health, emphasizing the need for psychiatrists to have a more holistic understanding of socio-urban phenomena.

**Objectives:**

1. To assess the clinical impact of urban alienation on mental health based on multi-disciplinary literature.
2. To particularly examine urban mental health concepts from outside psychiatry that may be relevant to clinical practice.
3. To identify possible strategies for integrating these interdisciplinary insights into daily practice and public mental health policy.

**Methods:**

We performed a multi-disciplinary literature review, analyzing studies from psychiatry, sociology urban studies, critical theory, and public health to evaluate the impact of social alienation on mental health. Special attention was paid to identifying both gaps and overlaps between disciplines.

**Results:**

We grouped findings into three major disciplinary areas: psychiatry, sociology, and urbanist theory. While each of these fields has unique histories and contributions, the literature lacks consistent integration among them. For clinicians in particular, there is significant conceptual language that has not yet entered the psychiatric lexicon. Across fields, it is noted that city-dwellers face alienation due to resource limitations, systemic issues, ideological pressures, and cultural barriers. Proposed solutions vary significantly based on discipline, including community-building activities, mental health support services, and inclusive urban planning.

**Conclusions:**

There is a breadth of research on cities, alienation, and mental health, and yet little integration of the disciplines. Addressing social alienation in urban environments requires psychiatric thought to move beyond isolated clinical interventions and toward collaborations with community organizations, policymakers, and urban planners. By aligning mental health expertise with the broader social and physical context, psychiatry can contribute to more meaningful, holistic interventions. Consequently, there is a pressing need for academic research bridging these fields, enabling more effective solutions that enhance community well-being in urban settings.

**Disclosure of Interest:**

None Declared

